# The influence of biological and lifestyle factors on circulating cell-free DNA in blood plasma

**DOI:** 10.7554/eLife.69679

**Published:** 2021-11-09

**Authors:** Nicole Laurencia Yuwono, Kristina Warton, Caroline Elizabeth Ford

**Affiliations:** 1 Gynaecological Cancer Research Group, Adult Cancer Program, Lowy Cancer Research Centre, Department of Obstetrics & Gynaecology, School of Women's and Children's Health, Faculty of Medicine & Health, University of New South Wales Sydney Australia; The Chinese University of Hong Kong Hong Kong; The Chinese University of Hong Kong Hong Kong

**Keywords:** Circulating cell-free DNA, cirDNA, biological, lifestyle, factors, plasma

## Abstract

Research and clinical use of circulating cell-free DNA (cirDNA) is expanding rapidly; however, there remain large gaps in our understanding of the influence of lifestyle and biological factors on the amount of cirDNA present in blood. Here, we review 66 individual studies of cirDNA levels and lifestyle and biological factors, including exercise (acute and chronic), alcohol consumption, occupational hazard exposure, smoking, body mass index, menstruation, hypertension, circadian rhythm, stress, biological sex and age. Despite technical and methodological inconsistences across studies, we identify acute exercise as a significant influence on cirDNA levels. Given the large increase in cirDNA induced by acute exercise, we recommend that controlling for physical activity prior to blood collection is routinely incorporated into study design when total cirDNA levels are of interest. We also highlight appropriate selection and complete reporting of laboratory protocols as important for improving the reproducibility cirDNA studies and ability to critically evaluate the results.

## Introduction

Circulating cell-free DNA (cirDNA) in blood has been extensively researched due to its potential as a biomarker across multiple settings including oncology, prenatal testing, toxicology, organ transplantation, and cardiovascular and autoimmune diseases. In cancer alone, cirDNA-related publications have increased exponentially (Trigg et al., 2018) with nearly 2000 publications solely in 2020. Considering the enormous investment and effort that has gone into the development of cirDNA biomarkers, understanding the biological and lifestyle factors that impact cirDNA levels is important for appropriate study design.

The release of cirDNA from cells into the blood is thought to be driven by apoptosis, which is closely linked to inflammation and cell damage. In this review, we aim to gather and summarise all published studies that have investigated an association between biological and lifestyle factors and cirDNA. By ‘biological factor‘, we refer to intrinsic physiological variables such as body mass index (BMI), menstruation, hypertension, circadian rhythm, stress, biological sex and age. By ‘lifestyle factor’, we refer to all external variables that involve individual choice, such as exercise, alcohol consumption, occupation and smoking. We have chosen to include only studies conducted on plasma and exclude all studies on serum as serum has been shown to be the less preferred substrate due to its propensity for leukocyte DNA contamination and dependence on processing time (Ammerlaan and Betsou, 2019; Trigg et al., 2018; Warton et al., 2014). The use of serum was the only exclusion criterion that we imposed. We included all identified plasma studies regardless of the sample size, cohort type (healthy or diseased) and laboratory methodology. The cohorts included, but were not limited to, people with cancer, hormone therapy-treated women and haemodialysis patients. In those studies, there are confounding factors related to pathology, and the cirDNA level is partly, if not primarily, influenced by disease-driven cirDNA changes.

In total, this review summarises the results from 66 individual studies published between January 2000 and January 2021 (Supplementary file 1). It is important to note that we did not aim to provide a statistical evaluation of the impacts of each factor on cirDNA level, but rather to create a summary of research endeavours in the field and to raise awareness of the need for large, adequately powered studies with appropriate technical protocols.

## 1 Biological factors

### 1.1 Body mass index

BMI, calculated by dividing weight (kg) by the square of height (m^2^), categorises an individual as underweight (<18.5), normal weight (18.5–24.9), overweight (25–29.9) or obese (>30). BMI, however, does not distinguish whether the weight is fat or muscle mass, hence its usage is not accurate for groups of people such as body builders and athletes.

Eight individual studies on cirDNA and BMI were identified, containing six healthy cohorts (n = 1464) and six cohorts that included individuals with various diseases and women undergoing hormone replacement therapy (HRT). Five of the studies conducted on healthy individuals, totalling to 1098 subjects, reported no relationship between cirDNA and BMI. They contained relatively large, combined numbers of healthy male and female subjects (763 males and 701 females), but most did not sex-disaggregate their data for analysis. A single study reported significantly higher cirDNA level with increasing BMI in a group of 366 healthy women (Jylhävä et al., 2014). Although this is the only study that discovered an association in healthy individuals, it has the strongest statistical power, albeit no information on how the cirDNA was processed (time to processing, number of spins and extraction method).

A positive correlation between BMI and cirDNA was found in 62 hepatitis-related liver fibrosis patients (46 males and 16 females) (Yan et al., 2018). However, in 30 gastric cancer patients (Kim et al., 2014), 218 women receiving oestrogen and/or progestin hormone treatment (Jylhävä et al., 2014), 58 non-alcoholic fatty liver disease patients (Karlas et al., 2017) and 113 type II diabetes (T2D) patients (Bryk et al., 2019) all did not show any relationship between BMI and cirDNA level. It may be that the effect of BMI on cirDNA is moderate and requires a large sample size (such as in the Jylhävä et al., 2014 study) for the effect to be observed. The positive correlation reported in the fibrosis patients may be due to BMI being associated with the disease itself (Cristina et al., 2018). If BMI is indeed linked to cirDNA, one proposed mechanism is that obesity disrupts the balance of the adipose tissue microenvironment, resulting in adipocytes undergoing apoptosis and/or necrosis and releasing cirDNA into the circulation (Nishimoto et al., 2016; Yan et al., 2018).

As discussed in Section 4, accurate cirDNA quantification is strongly dependent on extraction and measurement methods. Limiting discussion to studies that either measured cirDNA directly from plasma or used a dedicated extraction kit, two studies from the same group reported no relationship between BMI and cirDNA concentration in a total of 402 nonagenarians (Jylhävä et al., 2012; Jylhävä et al., 2013), while a single study with 62 liver fibrosis patients reported a positive relationship (Yan et al., 2018). Given the methodological limitations and the lack of consistent results, a definitive answer regarding the effect of BMI on cirDNA levels is still pending.

It is accepted that BMI is not an accurate reflection of obesity (Nuttall, 2015). The question then arises, if cirDNA is not correlated with BMI, is it correlated with fat mass itself? This was further supported by data from a cohort of 131 people (88 men and 44 women) investigated during medical examination. Higher visceral (abdominal) fat area (VFA ≥100 cm^2^), as determined by computed tomography, positively correlated with plasma single-stranded DNA concentration (Nishimoto et al., 2016). The authors further showed that cirDNA released from obese mice can bind to TLR9 receptors expressed by macrophages, leading to cell accumulation and assisting in causing chronic low inflammation associated with obesity (Nishimoto et al., 2016). In humans, although acute inflammation is associated with cirDNA increase, the correlation with chronic inflammation is inconclusive (Frank, 2016).

BMI is well known to be a risk factor for T2D (Al-Goblan et al., 2014; Bhowmik et al., 2015), a state where reduced insulin production or insulin resistance causes high blood glucose and ultimately leads to many physiological complications. Higher cirDNA has been found to be positively correlated with higher fasting glucose in 113 T2D patients (Bryk et al., 2019), but not in healthy female and male controls or females receiving oestrogen and/or progestin (Jylhävä et al., 2014). Furthermore, cirDNA is positively correlated with insulin levels in healthy women and women receiving oestrogen. but not in healthy men and women receiving oestrogen and progestin (Jylhävä et al., 2014).

### 1.2 Menstruation

The menstrual cycle is the regular succession of menstruation and ovulation in women. During the follicular phase, the endometrium thickens from approximately 5.4 mm to 9.2 mm (Tsuda et al., 2018). If fertilisation is absent, menstruation follows and is characterised by breakdown of the outer endometrial epithelium layer via apoptosis (Armstrong et al., 2017; Kokawa et al., 1996; Tabibzadeh, 1996) and extensive angiogenesis (Demir et al., 2010).

A single study examined the impact of menstruation on plasma cirDNA levels (Yuwono et al., 2021). Matched plasma samples from 40 women at the menstruating and non-menstruating phases of the cycle were compared, for total and endothelium-derived cirDNA, and no differences were observed. The strengths of this study were the adequate sample size (powered at 90% to detect 30% change), optimum cirDNA processing and good assay sensitivity.

These findings were unexpected given the extensive cell death and proliferation that occurs during the menstrual cycle. In addition to apoptosis occurring within the endometrium, leukocyte numbers increase during menstruation and contribute to the matrix metalloproteinases that break down the endometrial tissue (Maybin and Critchley, 2015); however, the eventual leukocyte cell death was not reflected by an increase in cirDNA amount. Shedding of apoptotic cell debris into the uterine cavity may account for lack of cirDNA increase during menstruation (Greystoke et al., 2013). This contrasts with scenarios like cancer or any diseases involving internal tissue damage, in which cell death occurs completely surrounded by stroma and therefore apoptotic or necrotic DNA is more likely to spill over into the circulation.

### 1.3 Hypertension

Hypertension, defined by World Health Organization (WHO), as having diastolic blood pressure (DBP) and systolic blood pressure (SBP) of ≥140 mm Hg and ≥90 mm Hg, respectively, can lead to stroke, kidney failure, blindness, rupture of blood vessels and cognitive impairment. The elevated pressure can cause endothelial cell DNA damage, inflammation and oxidative stress (Ranchoux et al., 2016), all of which could potentially result in cirDNA changes.

Five individual studies on cirDNA and hypertension were identified, containing four healthy cohorts (n = 1247) and five cohorts that included patients with various diseases and women undergoing HRT. Overall, conflicting findings were reported. No correlation between cirDNA levels and hypertension was found in 258 nonagenarians (Jylhävä et al., 2012), 218 women receiving oestrogen and/or progestin therapy (Jylhävä et al., 2014), 14 healthy women (Brodbeck et al., 2020) and 113 T2D patients (Bryk et al., 2019). The use of HRT has been shown to improve blood pressure (Butkevich et al., 2000; McCubbin et al., 2002) while the synergy between T2D and hypertension is well established (Lastra et al., 2014). By contrast, in 366 healthy women and 609 healthy men, cirDNA was positively correlated with SBP and/or DBP (Jylhävä et al., 2014). cirDNA was also positively correlated with SBP in 95 haemodialysis patients, 50 of whom were also diabetic (Jeong et al., 2015). Taking into account only studies that use cirDNA dedicated extraction kits or direct from plasma measurement (Brodbeck et al., 2020; Jeong et al., 2015; Jylhävä et al., 2012) retains a mixed picture of the impact of hypertension on cirDNA levels. It is likely that the effect, if any, is small, and may be mitigated by interaction with gender and HRT use.

Due to its global prevalence, the effects of hypertension on cirDNA are of interest. Future studies should include hypertensive people both with and without medication, as well as healthy controls. The studies should be disaggregated by sex and include subjects selected for the absence of other comorbidities and lifestyle variables that can potentially be confounding factors.

### 1.4 Circadian rhythm

The circadian rhythm, driven by the suprachiasmatic nucleus of the anterior hypothalamus of the brain, influences physiological profiles such as temperature, hormone levels and urine volume, as well as behaviour such as activity and sleep. Daytime is characterised by high body temperature, high urine volume, low melatonin and high cortisol, while the opposite occurs during night-time (Czeisler and Buxton, 2011; Silver and Schwartz, 2005). Disturbances in circadian rhythm lead to increased risk for a range of cancers including lung, colon, bladder and prostate cancers (Parent et al., 2012; Wendeu-Foyet et al., 2018).

Seven individual studies on cirDNA and circadian rhythm were identified, containing seven healthy cohorts (n = 73) and two cancer patient cohorts (colorectal and lung cancers). Overall, there is mixed evidence regarding circadian rhythm influence on cirDNA. No differences in cirDNA as a function of time were observed in a group of 11 healthy subjects measured at 7 AM, 12 PM and 5 PM (Korabecna et al., 2011) or in 4 healthy individuals measured every 2 hr and 45 min between 8:30 AM and 7:30 PM (Wagner et al., 2020). This lack of effect was also seen in 11 moderately trained men measured 11 times over 24 hr (Fatouros et al., 2010) as well as 1 healthy control and 10 colorectal cancer patients (stages I–IV) measured every 6 hr over a 24 hr interval (Tóth et al., 2017). In contrast, two studies reported a decrease in cirDNA levels between morning and evening in 13 and 4 subjects, respectively (Madsen et al., 2019; Meddeb et al., 2019).

Two studies implemented cirDNA tailored pre-analytical practice (short blood processing time, double centrifugation and appropriate cirDNA extraction kit), as well as limiting physical activity of study participants to minimise changes due to exercise. As described above, no circadian pattern was observed in four healthy subjects measured five times between 8:30 AM and 7:30 PM, with the experiment repeated on two separate days (Wagner et al., 2020). However, the study by Madsen and colleagues extended three hourly measurements from 9 AM to 9 PM in 13 healthy subjects and observed a gradual decrease that reached statistical significance at 9 PM (Madsen et al., 2019).

Two studies measured cirDNA fluctuations over short time intervals, specifically over 1 hr (Madsen et al., 2019) and every 5–15 min over 75 min (Brodbeck et al., 2020), both reporting no effect. Two studies compared cirDNA levels between days. There was no difference between days 1, 2 and 3 at 9 AM in either 33 healthy subjects or 10 lung cancer patients (Madsen et al., 2019) or in cirDNA measured 1 week apart (Wagner et al., 2020). All three aforementioned studies employed optimum cirDNA processing.

A relationship between cirDNA levels and circadian rhythm can be expected as multiple studies have shown cirDNA is primarily haematopoietic in origin (Lam et al., 2017; Lui et al., 2002; Moss et al., 2018; Snyder et al., 2016; Sun et al., 2015; Sun et al., 2019; Wong et al., 2016; Zheng et al., 2012) and haematopoietic cell numbers display a 24 hr circadian cycle (Ackermann et al., 2012; Pritchett and Reddy, 2015). On the other hand, the mechanism of cirDNA release into the bloodstream is mainly apoptosis (Jahr et al., 2001; Jiang et al., 2018; Jiang and Lo, 2016), and there is no evidence circadian rhythm influences apoptosis in healthy individuals, and only limited evidence in disease states (Gaddameedhi et al., 2015; Rabinovich-Nikitin et al., 2019; Wang et al., 2015).

The circadian rhythm studies do not report the time interval between waking and blood collection from subjects. Given the rapid clearance of cirDNA following exercise-induced increase, it is possible that cirDNA does change during sleep but returns to normal by the time a blood sample is taken for measurement. Furthermore, only two studies report what activity the participants were involved in between blood draws. In those studies, participants were told to minimise physical activity before blood draws (Madsen et al., 2019) or given access to recliner chairs and allowed to walk around freely (Wagner et al., 2020).

### 1.5 Psychological stress

Psychological stress burden has been linked to cirDNA changes through inflammation mechanisms (Liu et al., 2017) and DNA damage (Jenkins et al., 2014). Three individual studies on cirDNA and psychological stress were identified, containing two healthy cohorts and a cohort of women undergoing IVF treatment.

Brodbeck et al., 2020 took advantage of the stress created by venepuncture to look for a relationship between stress and cirDNA levels. The nervousness of study participants was quantified on a 0–10 scale. No relationship was observed between self-reported nervousness about the venepuncture procedure and cirDNA levels in 14 healthy women volunteers. However, the subjects might not be a true representative of a cohort experiencing a lot of stress since they volunteered for the venepuncture and were informed of the study procedure, and scaling, while easy and simple, is prone to bias.

Within a longer timeframe, Czamanski-Cohen et al., 2014 examined cirDNA in 14 women undergoing IVF treatment. Women who were offered 5–6 sessions with an experienced psychotherapist and implemented twice daily stress reduction techniques (n = 8) had significantly lower cirDNA levels at the end of the IVF cycle than women who received standard psychological care from IVF nursing staff (n = 6). The cirDNA was quantitated directly from plasma using a nucleic acid stain; however, pre-analytical procedures regarding blood tube type, blood processing time and centrifugation were not described.

Hummel et al., 2018 examined the acute effect of stress on cirDNA using the Trier social stress test – a validated and standardised acute laboratory stress challenge – in 20 healthy male sports science students. The test involved a free speech and an unanticipated math task performed in front of judges and a camera, all leading to consistently raised levels of cortisol. Immediately after the test cirDNA was found to have increased by about twofold, followed by a rapid drop back down to pre-test levels within 15 min. However, there was no correlation between cortisol and cirDNA (Hummel et al., 2018). With 20 paired samples, this study had the highest statistical power. The technical strengths were circadian rhythm control, with all blood sampling performed between 9 and 11 AM and optimum pre-analytical procedures (appropriate kit, immediate blood processing time, double centrifugation). Overall, there is some evidence for the impact of psychological stress on cirDNA.

### 1.6 Biological sex

Twenty-five individual studies on cirDNA and biological sex were identified. These included 12 cohorts of healthy subjects and 24 cohorts of patients with diagnosed disease (Table 1). In the healthy cohorts, 6/12 studies (n = 240) reported no difference while the remaining half (n = 1868) reported males having significantly higher cirDNA compared to females.

**Table 1.** Association of cirDNA with gender.

Authors (year)	Subject			Conclusion to gender differences on cirDNA amount
	Cohort	Male (n)	Female (n)	
Coulet et al., 2000	Head and neck squamous cell carcinoma patients (n = 117)	105	12	No effect
Sozzi et al., 2001	Control (n = 43)	NA	NA	No effect
	Lung cancer patients (n = 84)	72	12	No effect (p=0.403)
Tamkovich et al., 2005	Healthy volunteers (n = 35)	15	20	No effect
Zhong et al., 2007	Healthy adults (n = 54)	27	27	No effect
Lee et al., 2011	Lung cancer patients (n = 134)	13	121	No effect (p=0.947)
Beiter et al., 2011	Recreational runners (n = 53)	34	19	No effect
Jylhävä et al., 2012	Nonagenarians (n = 258)	62	196	Significantly higher in male (p=0.018)
Kim et al., 2014	Control (n = 34)	15	19	No effect (p=0.598)
	Gastric cancer patients (n = 30)	23	7	Significantly higher in female (p=0.01)
Spindler et al., 2014	Metastatic colorectal cancer patients (n = 86)	55	31	No effect (p=0.24)
Jylhävä et al., 2014	Finnish population	609–681	366–409	Significantly higher in male (p=0.00)
Jeong et al., 2015	Haemodialysis patients (n = 95)	NA	NA	No effect
	Diabetic haemodialysis patients (n = 50)	NA	NA	No effect (p=0.22)
Spindler et al., 2015	Metastatic colorectal cancer patients (n = 223)	126	97	No effect (p=0.1)
Chen et al., 2016	Stage I and II non-small cell lung cancer patients	33	25	No effect (p=0.318)
Hsieh et al., 2016	Oesophageal squamous cell carcinoma patients	70	11	No effect (p=0.315)
Karlas et al., 2017	Non-alcoholic fatty liver disease patients (n = 58)	32	26	90 bp fragment – no effect; 222 bp fragment – higher in female (p=0.0051)
Li et al., 2017	All lymphoma patients (n = 174)	107	67	No effect (p=0.769)
	Diffuse large B cell lymphoma (n = 98)	61	37	No effect (p=0.507)
Çayir et al., 2018	Control (n = 51)	28	23	Significantly higher in male
	Greenhouse workers (n = 72)	41	31	Significantly higher in male
Meddeb et al., 2019	Healthy individuals (n = 104)	62	42	Significantly higher in male (p=0.048)
	Colorectal cancer patients (n = 118)	68	50	No effect
van der Drift et al., 2010	Healthy controls (n = 21)	19	2	No effect
	Lung cancer patients (n = 46)	30	16	
Catarino et al., 2012	Healthy controls (n = 205)	78	127	Significantly higher in male (p<0.001)
	Lung cancer patients (n = 104)	86	20	No effect (p=0.123)
Wu et al., 2019	Newly diagnosed lymphoma patients (n = 60)	32	28	No effect (p=0.76)
	Treated lymphoma patients (n = 107)	59	48	No effect (p=0.4967)
Alghofaili et al., 2019	Healthy volunteers (n = 275)	124	151	Significantly higher in male (p=0.000103)
Caglar et al., 2020	Thyroiditis (n = 33)	NA	NA	No effect (p>0.7)
	Benign (n = 37)	NA	NA	No effect (p=0.054)
	Malignant (n = 30)	NA	NA	No effect
	All thyroid patients (n = 100)	NA	NA	No effect (p=0.08)
Bryk et al., 2019	T2D patients (n = 111)	NA	NA	No effect (p=0.51)
Bu et al., 2020	Gastric cancer patients (n = 61)	41	20	No effect

T2D: type II diabetes.

In patient cohorts and workers exposed to occupational hazards, most studies (21/24) report no differences in cirDNA between males and females. A single study of 72 greenhouse workers reported higher cirDNA level in males (Çayir et al., 2018), but the remaining two patient studies reported higher cirDNA in females (Karlas et al., 2017; Kim et al., 2014). However, of these, one analysis, conducted in 30 gastric patients, had imbalanced sample numbers with 23 males and only 7 females (Kim et al., 2014), while the other only saw the association with long cirDNA fragments (222 bp), with no effect on short fragments (90 bp) (Karlas et al., 2017).

Considering only studies in healthy volunteers that either measured cirDNA directly from plasma or used a dedicated extraction kit, higher cirDNA levels in males relative to females were measured in three cohorts (total of 584 subjects) (Alghofaili et al., 2019; Çayir et al., 2018; Jylhävä et al., 2012), while no effect was observed in a single cohort of 43 subjects (Sozzi et al., 2001). If sex plays a role, the evidence favours males having higher cirDNA than females. However, the effect of sex appears fairly small, and may be confounded by lifestyle differences between males and females. Carefully controlled studies with large sample numbers, combined with meta-analyses, will be helpful in quantifying the association.

### 1.7 Age

Twenty-nine individual studies on cirDNA and age were identified. These contained 45 cohorts, with 19 groups of healthy individuals and 26 of patients with diagnosed disease and women undergoing HRT (Table 2). Amongst the healthy cohorts, 14/19 reported no impact of age on cirDNA. Of those that showed a significant difference (5/19), all found that cirDNA is positively correlated with age (Jylhävä et al., 2011; Jylhävä et al., 2014; Jylhävä et al., 2013; Meddeb et al., 2019; Zhong et al., 2007); however, a significant effect was observed in women only in three out of the five studies (Jylhävä et al., 2011; Jylhävä et al., 2014; Zhong et al., 2007). Three of the five studies showing a relationship between age and cirDNA levels in healthy cohorts are from a single research group (Jylhävä et al., 2011; Jylhävä et al., 2014; Jylhävä et al., 2013). While the laboratory methodology is comparable to other published work, two of the studies were unique in investigating cohorts with a large age difference, rather than looking for a continuous effect. cirDNA was compared between two distinct cohorts (19–37 years versus nonagenarians) (Jylhävä et al., 2011; Jylhävä et al., 2013), both mostly women. Since the age difference is large, it is possible the effect is apparent even when the sample size is modest (11 controls and 12 nonagenarians) (Jylhävä et al., 2011). Another publication by the same group reported an age effect in healthy women (average age 60.48 years ± 8.98) and had a substantial sample size of 366 (Jylhävä et al., 2014).

**Table 2.** Association of cirDNA with age.

Authors (year)	Subject	Age analysed	Conclusion to age effect on cirDNA amount
Sozzi et al., 2001	Control (n = 43)	-	No effect
	Lung cancer patients (n = 84)	39–59 (n = 31) vs. 60–69 (n = 34) vs. ≥70 (n = 19)	
Sozzi et al., 2003	Lung cancer patients(n = 100)Mean age: 65.1 ± 8.9	≤60 vs. 61–71 vs. ≥72	Significantly higher with increasing age
Tamkovich et al., 2005	Healthy participants (n = 35 [15 M + 20 W])	18–53	No effect
Zhong et al., 2007	Healthy adults	20–40 vs. 41–60 vs. >60	No effect (men and women mixed)Significantly higher cirDNA for >60 years old compared to 20–40 and 41–60 groups in women only
Lee et al., 2011	Lung cancer patients (n = 134)	≤65 (n = 108) vs. >65 (n = 26)	No effect (p=0.333)
Beiter et al., 2011	Recreational runners (n = 53)Mean age: 34.8	17–60 years	No effect
Jylhävä et al., 2011	Control (n = 11, females, 22–37 years old) vs. nonagenarians (n = 12, females, born 1917)		Significantly higher in elderly (p=0.035, < 0.001, 0.015)^*^
Jylhävä et al., 2013	Young controls (n = 30 [9 M + 21 W], aged 19–30 years old) vs. nonagenarians (n = 144 [43 M + 101 W], aged ≥90 years old)		Significantly higher in nonagenarian group (p=0.002)
Kim et al., 2014	Control (n = 34)Mean age = 63.79 ± 6.76 years	<65 vs. ≥65	No effect
	Gastric cancer patientsMean age = 66.72 ± 13.16 years		Significantly higher with increasing age
Spindler et al., 2014	Metastatic colorectal cancer patients (n = 86)Median age: 66 (37–83)	<66 (n = 43) vs. >66 (n = 43)	No effect
Jylhävä et al., 2014	Women (n = 366–409, mean age 60.48 [8.98])		Significantly higher with increasing age (p=0.002)
	Women (oestrogen HRT user, n = 131–148, mean age 58.57 [6.88])		No effect (p=0.391)
	Women (oestrogen + progestin HRT user, n = 87–98, mean age 57.23 [6.39])		No effect (p=0.869)
	Men (n = 609–681), mean age (58.31 [7.91])		No effect (p=0712)
Breitbach et al., 2014a	Male athletes (n = 26 [13 handball players + 13 triathletes])Mean age 24.7 (3.1)		No effect
Jeong et al., 2015	Haemodialysis patients (n = 95)	58 ± 1.5	No effect
	Haemodialysis patients (n = 95)	58 ± 1.5	No effect (p=0.80)
	Diabetic haemodialysis patients (n = 50)	66.4 ± 1.8	No effect (p=0.93)
Spindler et al., 2015	Metastatic colorectal cancer patients (n = 223)Median age: 63 (35-82) years old	≤63 (n = 119) vs. >63 (n = 104)	No effect (p=0.39)
Korzeneva et al., 2015	Average age for all groups: 48.5 ± 16.3 years		
	Never-exposed control group (n = 109)	*21–86	No effect (p=0.13)
	Chronic gamma-neutron radiation-exposed group (n = 88)	*26–79	No effect (p=0.6)
	Chronic tritium β-radiation-exposed group (n = 88)	*20–80	No effect (p=0.06)
	Never-exposed control group (n = 109)	<65 years old vs. ≥65 years old	Significantly higher with increasing age
	Chronic gamma-neutron radiation-exposed group (n = 88)	<65 years old vs. ≥65 years old	Significantly lower with increasing age
	Chronic tritium β-radiation-exposed group (n = 88)	<65 years old vs. ≥65 years old	No effect
Hsieh et al., 2016	Oesophageal squamous cell carcinoma patients (n = 81 [70 M + 11 F])	<60 (N = 43) vs. >60 (N = 38)	No effect (p=0.588)
Tosevska et al., 2016	Institutionalised elderly aged 65–98 (n = 105)	65–98	No effect
Karlas et al., 2017	Non-alcoholic fatty liver disease patients (n = 58)	Age (mean age 62.1 ± 11 years old)	No effect
Li et al., 2017	All lymphoma patients (n = 174)	≤60 (N = 117) vs. ≥60 (N = 57)	No effect (p=0.414)
	Diffuse large B cell lymphoma (n = 98)	≤60 (N = 61) vs. ≥60 (N = 37)	No effect (p=0.668)
Beranek et al., 2017	Exacerbated psoriasis vulgaris patients (n = 28 [15 M + 13 W])	18–69 (median age 50)	No effect
Teo et al., 2019	Young (n = 3) vs. elderly (n = 3) vs. healthy centenarians (n = 3) vs. unhealthy centenarians (n = 3)		No effect
Meddeb et al., 2019	Healthy individuals (n = 104)Age range: 18–69	<47 (n = 52) vs. ≥47 (n = 52)	Significantly higher with increasing age (p=0.009)
	Healthy individuals (n = 104)	Young (n = 79) vs. older (n = 25)†	Significantly higher with increasing age (p=0.0026)
	Colorectal cancer patients (n = 118)Age range: 22–91	<65 (n = 52) vs. ≥65 (n = 66)	No effect
	Colorectal cancer patients (n = 118)	Young (n = 25) vs. older (n = 93)^†^	No effect (p=0.913)
van der Drift et al., 2010	Healthy controls	<60 vs. ≥60	No effect (p=0.43)
	Lung cancer	<60 vs. ≥60	No effect (p=0.25)
Catarino et al., 2012	Healthy controls (n = 205)	<64 vs. ≥64	No effect (p=0.342)
	Lung cancer patients (n = 104)	<64 vs. ≥64	No effect (p=0.614)
Wu et al., 2019	Newly diagnosed lymphoma patients (n = 60)	<60 vs. ≥60	No effect (p=0.4041)
	Treated lymphoma patients (n = 107)	<60 vs. ≥60	No effect (p=0.3127)
Alghofaili et al., 2019	Healthy volunteers (n = 275)	Correlation plot (0–57 years old; median 27 years old)	No effect (*r* = –0.09)
Caglar et al., 2020	Thyroiditis (n = 33)	37.6 ± 10.9	No effect
	Benign (n = 37)	54.1 ± 13.1	No effect
	Malignant (n = 30)	47.8 ± 11.9	No effect
	All thyroid patients (n = 100)		Significant positive correlation (p<0.05)
Bryk et al., 2019	T2D patients		No effect (p=0.63)
Bu et al., 2020	Gastric cancer patients (n = 61)	40–83	No effect (p=0.323 and p=0.280)^‡^

* Three different extraction kits.

† Using same cutoff for both healthy and cancer cohort as the median age (56) of all individuals tested.

‡ Two different extraction kits.

HRT: hormone replacement therapy; T2D: type II diabetes.

Considering only studies with healthy cohorts and cirDNA-tailored methodology, two studies with a total of 197 subjects observed a relationship between cirDNA levels and age (Jylhävä et al., 2011; Jylhävä et al., 2013), while five studies with a total of 461 subjects reported no relationship (Alghofaili et al., 2019; Breitbach et al., 2014a; Sozzi et al., 2001; Teo et al., 2019; Tosevska et al., 2016). However, as discussed below, comparisons between individual studies are difficult due to the different age ranges of subjects included, for example, very young versus very old (Jylhävä et al., 2011; Jylhävä et al., 2013), compared to studies with a smaller age range (Breitbach et al., 2014a).

In a comprehensive study of the tissue sources of cirDNA conducted by Moss et al., 2018, the sources of cirDNA were unchanged with age; however, older people had higher cirDNA levels. The consistent tissue profile could indicate decreased capacity of cirDNA clearance rather than an increase in apoptosis of specific tissues (Moss et al., 2018). Similarly, Teo et al., 2019 found little change between young people (25 ± 0.5 years old) and healthy centenarians but found larger fluctuations among healthy older subjects (71 ± 1.6 years old) and unhealthy centenarians, including decreased pituitary gland and increased tibial artery cirDNA.

In the patient cohorts, 23/26 observed no age effect, while in three studies comprising 100 thyroid-related pathology patients (Caglar et al., 2020), 30 gastric cancer patients (Kim et al., 2014) and 100 lung cancer patients (Sozzi et al., 2003), a positive correlation with age was seen.

Comparing the effect of age across different studies is difficult for several reasons. Firstly, the way the subjects are categorised, and therefore analysed, varies between the studies. Some studies use specific age cutoffs (younger than or older than a certain age) to split their cohorts into two or three continuous groups. For example, Korzeneva et al., 2015 found no effect in either 109 healthy controls or 88 people chronically exposed to gamma-neutron radiation, unless the cohorts were split into two age groups, which resulted in significantly higher and lower cirDNA with increasing age, respectively. Some studies compared two groups with distinct ages such as Jylhävä et al., 2011, where a young age group (22–37 years old) is compared to nonagenarians. Some studies do not split their subjects but perform a correlation statistical analysis (Table 2). Overall, based on the evidence, the effect of age on cirDNA levels appears small.

## 2 Lifestyle factors

### 2.1 Exercise

Of all variables reviewed, exercise holds the most evidence to support its effect on cirDNA levels. A large and robust increase in cirDNA levels is associated with acute exercise, while less consistent changes are reported with chronic exercise (reviewed by Breitbach et al., 2012 and Vittori et al., 2019). Studies of exercise and cirDNA levels are summarised in Table 3.

**Table 3.** cirDNA measurements in acute exercise.

Authors (year)	Setting	Subject	CirDNA measurement time points
Atamaniuk et al., 2004	Race (did not specify duration and distance)	Healthy half-marathon runners (n = 25 [12 M + 13 F])	Before the race, immediately after race, 2 hr post-race
Margeli et al., 2005	246 km ultra-marathon	Healthy males (n = 15)	Pre-race, post-race (within 15 min), post-race (48 hr)
Atamaniuk et al., 2008	6 hr race	Experienced ultra-marathon runners (n = 14 [9 M + 5 F])	Pre-race, post-race, post-race (2 hr), post-race (24 hr)
Fatouros et al., 2010	Control (rest): remain seated/lying in the labExercise: 45 min treadmill run followed by increase in speed until exhaustion	Moderately trained men (n = 11)	Pre-exercise, post-exercise, post-exercise (0.5 hr, 1, 2, 3, 4, 5, 6, 8, 10, 24 hr)
Atamaniuk et al., 2010	Six sets of six weightlifting exercise	Male competitive weightlifters (n = 12)	Pre-exercise, post-exercise (immediately after), post-exercise (2 hr)
Beiter et al., 2011	Public 10 km cross-country interval run	Recreational runners (n = 53 [34 M + 19 W])	Pre-exercise, immediately after
	Incremental test on treadmill (until exhaustion)	Well-trained male athletes (n = 9)	Pre-exercise, immediately after, post-exercise (30 min)
	Strenuous treadmill until exhaustion	Well-trained endurance male athlete (n = 1), moderately trained female participant (n = 1), well-trained recreational male runner (n = 1)	Pre-exercise, mid-exercise (3, 6, 9, 12, 15 min), post-exercise (5, 10, 15, 20, 30 min)
de Sousa et al., 2012	Overload training programme (day 1–8) then 10 × 800 m sprints on day 9	Highly competitive male endurance runners (n = 24)	Day 1, day 9 (pre-exercise [–140 min], post-exercise [immediate, 80 min])
Breitbach et al., 2014a	Treadmill until exhaustion (average 17.9 min)	Male athletes (n = 26 [13 handball players + 13 triathletes])	Pre-exercise, post-exercise
Beiter et al., 2014	Increment treadmill until exhaustion	Well-trained male athletes (n = 6)	Pre-exercise, post-exercise (immediately), post-exercise (30 min)
	High-intensity 60 min cycling	Untrained males (n = 6)	Pre-exercise, post-exercise (immediately), post-exercise (3 hr)
		Regularly endurance trained males (n = 6)	Pre-exercise, post-exercise (immediately), post-exercise (3 hr)
Breitbach et al., 2014b	10 km relay race	Recreational runners (n = 10 [6 M + 4 F])	Pre-exercise, post-exercise
Tug et al., 2015	Incremental treadmill test	Healthy male controls (n = 3)	Pre-exercise, post-exercise (immediately), post-exercise (90 min)
		Healthy female controls (n = 3)	
		Sex-mismatched haematopoietic stem cell transplantation patients (n = 5 females with male donors)	
		Sex-mismatched haematopoietic stem cell transplantation patients (n = 2 males with female donors)	
Helmig et al., 2015	Incremental treadmill test until exhaustion	Physically active men (n = 5)	Pre-exercise, post-exercise (immediately after, 10, 30, 90 min)
Frühbeis et al., 2015	Increment cycling test until exhaustion	Physically active male (more than 3 hr/week) tested twice (n = 1)	Pre-exercise, mid-exercise (3, 6, 9, 12, 15, 18, 21 min), post-exercise (immediately after, 10, 30, 90 min)
Tug et al., 2017b	Acute strength exercise (whole-body exercises, deadlifts, squats and muscle-targeted exercises)	Regular strength trained men (n = 16)	12th, 13th, 14th, 15th, 16th exercise
	High-intensity training	n = 5/16	Before first exercise, after last exercise
	Differential training	n = 5/16	Before first exercise, after last exercise
	Conservation training	n = 6/16	Before first exercise, after last exercise
Tug et al., 2017a	Incremental bicycle exercise until exhaustion	Competitive male cyclists (n = 11)	Pre-exercise, post-exercise, post-exercise (90 min)
Stawski et al., 2017	Treadmill until exhaustion	Averaged-trained men (n = 11)	Pre- and post-1st bout, 2nd bout and 3rd bout of exercise
Haller et al., 2017	Stepwise increment running test until exhaustion	Athletes (n = 14 [7 M + 7 W])	Pre-exercise, mid-exercise (3, 6, 9, 12, 15, 18, 21 min), post-exercise (15, 30 min)
	40 min endurance run at 9.6 km/hr	Athletes (n = 13 [7 M + 6 W])	Pre-exercise, post-exercise
Hummel et al., 2018	Treadmill until exhaustion	Male students of sports science (n = 20)	Pre-exercise (–2 min), post-exercise (2, 15, 30, 40 min)
Haller et al., 2018	5 × 40 m sprints (5.94 ± 0.50 s)	Healthy subjects (n = 9 [7 M + 2 F])	Pre-exercise, post-exercise
	Treadmill test	Male football players playing more than 70 min in game and participated in treadmill test (n = 10)	Pre-exercise, post-exercise
	Season football game		Pre-exercise, post-exercise
Ferrandi et al., 2018	High-intensity interval exercise (30 min)	Healthy male subjects (n = 14 [seven normal weight and seven obese])	Pre-exercise, post-exercise, post-exercise (1 hr)
Ohlsson et al., 2020	Cycling until maximal heart rate	Healthy volunteers (n = 8 [4 M and 4 W])	Pre-exercise, sub-max load, max load, post-exercise (30, 90 min)
Mavropalias et al., 2021	Eccentric cycling	Men unaccustomed to eccentric exercise (n = 20)	Pre-exercise, post-exercise, post-exercise (24, 48, 72 hr)

### 2.1.1 Acute exercise

Twenty-two individual studies on cirDNA and acute exercise were identified. In acute exercise, all studies reviewed reported a significant increase in cirDNA during exercise and a decline to pre-exercise levels after cessation. This applied in both resistance and endurance exercise. Resistance exercise works to enhance the strength and build skeletal muscles by repeatedly overcoming resistance force. Two studies in this category reported a 1.4- to 1.7-fold and 3-fold increase in cirDNA after performing strength exercise (Tug et al., 2017b) and weightlifting exercises (Atamaniuk et al., 2010), respectively.

Endurance exercise is activity that increases breathing and heart rate, such as walking, jogging and cycling. Five studies used cycling regime to test their participants. All reported a significant increase in cirDNA ranging from 1.6- to 7.82-fold (Beiter et al., 2014; Frühbeis et al., 2015; Mavropalias et al., 2021; Ohlsson et al., 2020; Tug et al., 2017a). All remaining endurance studies subjected participants to running, ranging from 800 m sprints to 246 km ultra-marathons, with the majority using ‘treadmill test until exhaustion’ (Table 3). All studies reported the same transient rise and fall cirDNA pattern, spanning from 1.6- to 18.6-fold increase from pre-exercise level.

Endurance exercise appears to induce a larger cirDNA response than resistance exercise. Nearly half (10/25) of the endurance exercises reported cirDNA increases ≥9-fold, and the maximum reported increase was 18.6-fold (Atamaniuk et al., 2004). In contrast, amongst the resistance studies, the highest difference was a threefold increase in cirDNA after weightlifting (Atamaniuk et al., 2010). However, this needs further investigation as the number of studies examining resistance exercise was limited to 2.

#### Duration and intensity of acute exercise

There is some evidence for a positive relationship between duration of exercise and fold change in cirDNA. Mavropalias and colleagues investigated the effect of exercise intensity by splitting their cohort of 20 men into low- or high-intensity eccentric cycling groups. High-intensity cycling induced a significantly higher cirDNA increase in plasma than low-intensity cycling (Mavropalias et al., 2021). Furthermore, five 40 m sprint sessions with long rest intervals (5 min) caused a lower rise in cirDNA than five sprint sessions with short rest intervals (1 min) (Haller et al., 2018), and 24 competitive endurance runners reported a >9.8-fold increase after ten 800 m sprints (de Sousa et al., 2012), while 14 ultra-marathon runners experienced roughly an 18-fold increase after a 6 hr race, with a level above baseline persisting for at least 2 hr (Atamaniuk et al., 2008). Additionally, Haller et al., 2017 and Haller et al., 2018 showed a positive association between duration and distance covered and cirDNA increase during a low-intensity running exercise (Haller et al., 2017) and a football game (Haller et al., 2018), respectively.

#### Kinetics of cirDNA change during acute exercise

The kinetics of cirDNA during exercise indicate rapid initiation of clearance, with cirDNA peaking either immediately (Fatouros et al., 2010; Haller et al., 2017), 15 min (Hummel et al., 2018) or 30 min (Ohlsson et al., 2020) after exercise. The time required for cirDNA levels to return to baseline is not well defined as studies have reported both elevated (Ohlsson et al., 2020) and a return to baseline levels (Frühbeis et al., 2015) 1.5 hr post-cycling exercise.

Grouping participant results in kinetic studies might not be appropriate due to interindividual differences in cirDNA clearance. Beiter et al., 2011 examined the variation in cirDNA kinetics between individuals, subjecting three people to a treadmill test until exhaustion: one well-trained male endurance athlete, one moderately trained female participant and one well-trained recreational male runner. Although all three had the same general transient pattern, the peak occurred at different times and the decline time also varied (Beiter et al., 2011).

Since 55% of cirDNA comes from leukocytes (Moss et al., 2018), it is possible that the kinetics of leukocytes during exercise influence the kinetics of cirDNA. Increases in leukocyte number during exercise have been observed in several studies (de Sousa et al., 2012; Ohlsson et al., 2020; Tug et al., 2017a). For example, during a cycling exercise, the number of leukocytes peaked at maximal workload while the cirDNA level peaked 30 min after the cessation of exercise (Ohlsson et al., 2020). This burst of cirDNA occurring just after the leukocyte peak could be the result of leukocyte apoptosis during exercise (Krüger and Mooren, 2014). Additionally, circulating DNases may play a part in the clearance of cirDNA following exercise. Beiter et al., 2014 found that DNase activity follows the kinetic pattern of cirDNA, rising by 15-fold immediately after a treadmill test and decreasing to pre-exercise activity levels after 30 min. Moreover, exercise changes blood and plasma volume, which might also affect total cirDNA quantitation (Fellmann, 1992; Kawabata et al., 2004).

#### Individual fitness

Individual fitness levels appear to have little impact on cirDNA increase and clearance rates. Beiter et al., 2014 investigated the difference in cirDNA changes between untrained and endurance-trained males after 60 min of high-intensity cycling. Both groups had similar pre-exercise cirDNA levels, and the untrained and trained group had 6.9- and 4.5-fold increases, respectively. For both groups, cirDNA returned to normal after 3 hr and there was no statistically significance difference between the two groups in the way the cirDNA level responded to exercise.

#### Sex differences in cirDNA change during exercise

It is difficult to conclude if there are any differences in the way cirDNA levels respond to exercise in men compared to women. Half of the research studies conducted (11/21) involved male subjects only. Eight studies had a mix of males and females with equal or near equal representation, while two consisted predominantly of male subjects (Table 3). The lack of equal representation is an ongoing issue in the field of sports science, including in cirDNA-related exercise studies, which we highlighted in our recent publication (Hagstrom et al., 2021). Furthermore, in all studies except one, the data on male and female participants were combined, making delineation of sex differences not possible.

### 2.1.2 Chronic exercise

Six individual studies on cirDNA and chronic exercise were identified, far fewer studies compared to acute exercise. Overall, it appears to have less robust effects than acute exercise. The chronic exercise studies ranged in duration from 8 days (de Sousa et al., 2012) to 6 months (Tosevska et al., 2016), and the predominant exercise examined was resistance-type (4/6 studies) rather than endurance-type (2/6 studies). In all studies, blood samples were collected at least 1 day following cessation of exercise to avoid the confounding effect of acute exercise.

No change in cirDNA levels was observed following a chronic intense running programme in male endurance runners (de Sousa et al., 2012), resistance training in 23 (control group) and 27 (resistance training) elderly participants (Tosevska et al., 2016) or 8 weightlifters (Gentles et al., 2017). In contrast, two studies reported a significant cirDNA increase after performing resistance multi-joint exercises (Fatouros et al., 2006) and a range of strength exercises (Tug et al., 2017b) in 17 recreationally and 16 regularly trained men, respectively. One study of male soccer players observed a significant mid-season cirDNA increase only in players who started games in autumn season (A. Gentles et al., 2015).

Inconsistent effects of chronic exercise intensity are also reported. Fatouros et al., 2006 subjected participants to four 3-week training blocks of varying intensity and found that cirDNA peaked during the highest volume of training and fell as training intensity was decreased. This is opposed by the finding of Tug et al., 2017b that neither low- nor high-intensity acute strength training showed significant association with cirDNA levels. In addition, the frequency of habitual exercise (ranging from 0 to 7 times a week) had no association with cirDNA levels in 14 healthy women (Brodbeck et al., 2020).

### 2.1.3 Origin of cirDNA increase during exercise

The increase in cirDNA during exercise has been attributed to apoptosis due to the ladder pattern of DNA fragments (Thierry et al., 2016). This pattern is clearly shown in plasma collected before an ultra-marathon race, immediately after, and 2, and 4 hr after the race (Atamaniuk et al., 2008).

Sex-mismatched haematopoietic stem cell transplantation provides a means to determine whether the exercise-induced cirDNA increase originates from haematopoietic lineage cells. In one study, male and female patients with sex-mismatched transplant donors performed incremental treadmill exercise until exhaustion and their total and Y-chromosome cirDNA levels were measured. The results showed that most of the increase in cirDNA is from haematopoietic cells and not from the liver (Tug et al., 2015). Further evidence supporting exercise-induced leukocyte apoptosis is the significant decrease of Bcl2 mRNA expression immediately after exercise, while Bax and Bad mRNA levels increased slightly (Atamaniuk et al., 2008). However, post-exercise blood smears and immunohistochemistry indicated that the damaged cells are neutrophils undergoing a phenomenon termed neutrophil extracellular traps (NETs). In another study, cirDNA was positively associated with myeloperoxidase which is abundant in primary granules of neutrophils (Beiter et al., 2014), providing further evidence for the role of neutrophils in the origin of cirDNA.

Another clear candidate for a cirDNA source during exercise is skeletal muscle; however, only one study to date has specifically measured muscle-derived cirDNA in this setting. Surprisingly, no increase in skeletal muscle-derived cirDNA, as identified via the methylated HOXD4 promoter, was observed after exercise. The authors only analysed 5 out of 20 men recruited in this tissue-origin investigation (Mavropalias et al., 2021). Muscle tissue does not normally contribute to cirDNA (Moss et al., 2018); however, biochemical markers of tissue damage such as creatinine kinase and myoglobin have been reported (Nédélec et al., 2012; Thorpe and Sunderland, 2012). More research is required to validate the result by Mavropalias et al., 2021 and also to determine other possible sources of cirDNA in exercise.

### 2.2 Alcohol

There is a strong association between alcohol consumption and increased risk of cancers such as liver, breast, oesophageal and pancreatic cancer (Ratna and Mandrekar, 2017). Due to the DNA-damaging properties of alcohol, it has been hypothesised that cirDNA levels increase as a result of alcohol-induced apoptosis (Brooks and Theruvathu, 2005).

Two studies measured association between alcohol consumption and plasma cirDNA levels (Hsieh et al., 2016; Kim et al., 2014) and found no correlation between alcohol intake and cirDNA concentration in either 24 healthy subjects, 23 gastric cancer patients (Kim et al., 2014) or 81 oesophageal cancer patients (Hsieh et al., 2016). Their technical limitations include missing methodological reporting, non-specialised cirDNA extraction kit and no clear specification of alcohol intake used for ‘moderate-severe’ categorisation (Kim et al., 2014) as well as the grouping of ‘social drinker’ and ‘had quit drinking for >5 years’ subjects into a single cohort (Hsieh et al., 2016). Combining data from ‘social drinkers’ with ‘non-drinkers’ can obscure physiological effects since people with long-term abstinence can have different health characteristics to current drinkers (Fein et al., 2006; Sullivan et al., 2010).

In both studies, alcohol consumption was recorded as a part of investigating plasma cirDNA in the context of cancer. A more focused study that utilises protocols tailored to cirDNA measurements to confirm the effect of alcohol on cirDNA is needed. Both acute and chronic effects of alcohol should be investigated with clear information on alcohol intake amounts and duration. Recall bias is also a major issue in self-count-based studies which leads to under-reporting particularly for heavy and non-routine drinking (Boniface et al., 2014; Cherpitel et al., 2018).

### 2.3 Occupational hazard exposure

Two studies on cirDNA and occupational hazard exposure were identified. One study examined ionising radiation in 176 people working at nuclear sites (Korzeneva et al., 2015) and the second study examined pesticide exposure in 72 greenhouse workers (Çayir et al., 2018). cirDNA was significantly lower in subjects exposed to gamma-neutron radiation (n = 88) and chronic tritium β-radiation (n = 88) compared to a never-exposed group (n = 109). The authors postulated that this decrease may be due to an elevated level of DNase I in the exposed group (Korzeneva et al., 2015). The second study measured DNA directly from plasma and found that greenhouse workers exposed to pesticides for 5–15 years have near double cirDNA levels compared to non-exposed controls and showed a positive correlation between cirDNA concentration and pesticide exposure interval (Çayir et al., 2018). The strengths of the latter study include accounting for smoking, ethnic, social and cultural differences, as well as black tea consumption between exposed and control groups, but information on blood processing time was lacking and a second plasma centrifugation was omitted. Both studies have moderate combined sample sizes (160 control and 248 exposed subjects total). No analysis of the specific tissues that contribute to the increase in cirDNA concentration was carried out in either study.

### 2.4 Smoking

Cigarette smoke has potent DNA-damaging constituents (Pfeifer et al., 2002; Weng et al., 2018; Zhao et al., 2009); therefore, it is surprising that the documented effect of smoking on cirDNA is scarce. Smoking status is routinely collected in clinical records, especially in the context of cancer.

Ten individual studies on cirDNA and smoking were identified, containing 6 healthy cohorts and 13 cohorts that include cancer patients, women undergoing IVF treatment and greenhouse workers. Four out of six studies that included healthy cohorts reported no effect of smoking on cirDNA levels in a combined sample size of 444 (Catarino et al., 2012; Kim et al., 2014; Sozzi et al., 2003; Yoon et al., 2009). The remaining two studies reported opposing results – higher cirDNA level in former smokers (n = 8) versus current smokers (n = 12) in a study that used a non-specialised kit (van der Drift et al., 2010) and, conversely, higher cirDNA levels in current smokers (n = 13) versus non-smokers (n = 38) quantitated directly from plasma and measured within a cohort that did not segregate the pesticide-exposed greenhouse workers and unexposed controls (Çayir et al., 2018).

Amongst the cancer studies, four lung cancer (Catarino et al., 2012; Sozzi et al., 2003; van der Drift et al., 2010; Yoon et al., 2009), one oesophageal squamous cell cancer (Hsieh et al., 2016) and one thyroid cancer (Caglar et al., 2020) study reported no effect of smoking on cirDNA levels. One gastric cancer study reported significantly lower cirDNA amount in current smokers versus former smokers and non-smokers (Kim et al., 2014). The authors suggested that this may be because the cancer type that occurs in the aged, female, non-smoking cohort is more aggressive than others.

There were no cirDNA differences between former and current smokers versus non-smokers in a group of 37 women undergoing IVF-embryo transfer treatment (Czamanski-Cohen et al., 2013) or in T2D patients (Bryk et al., 2019). cirDNA was not associated with number of packs per year (Yoon et al., 2009), or smoking duration; however, smokers did have more extreme elevated values among cancer-free individuals, possibly reflecting smoking-associated health problems (Sozzi et al., 2003).

One limitation across the published studies is the imbalance of non-smoking and smoking cohorts. For example, in the study conducted by Sozzi et al., 2003, there were 7 never smokers, and 82 and 65 smokers in the healthy controls and lung cancer patients, respectively. This can be controlled for in future investigations, as well as matching the groups in age and gender. Due to the self-reported nature of smoking data and the tendency for underestimation (Connor Gorber et al., 2009), large cohort numbers would also be important.

## 3 Other factors

Many other variables have been investigated in smaller individual studies. No effect on cirDNA has been noted in studies examining height (Breitbach et al., 2014a), spring versus autumn season (Çayir et al., 2018), number of blood volunteering times (Zhong et al., 2007), history of betel nut chewing (Hsieh et al., 2016) or haematocrit or cannula placement pain (Brodbeck et al., 2020).

There is evidence that meal-derived DNA is present in the circulation. This DNA is mostly intact (>10 kbp) (Spisák et al., 2013). However, whether this impacts measurably on the cirDNA concentration remains unclear. Only a single study, which found no relationship between cirDNA and time since last food intake, has been published (Meddeb et al., 2019), and the effect of specific foods has not been examined.

## 4. Study methodology

**Figure 1.**
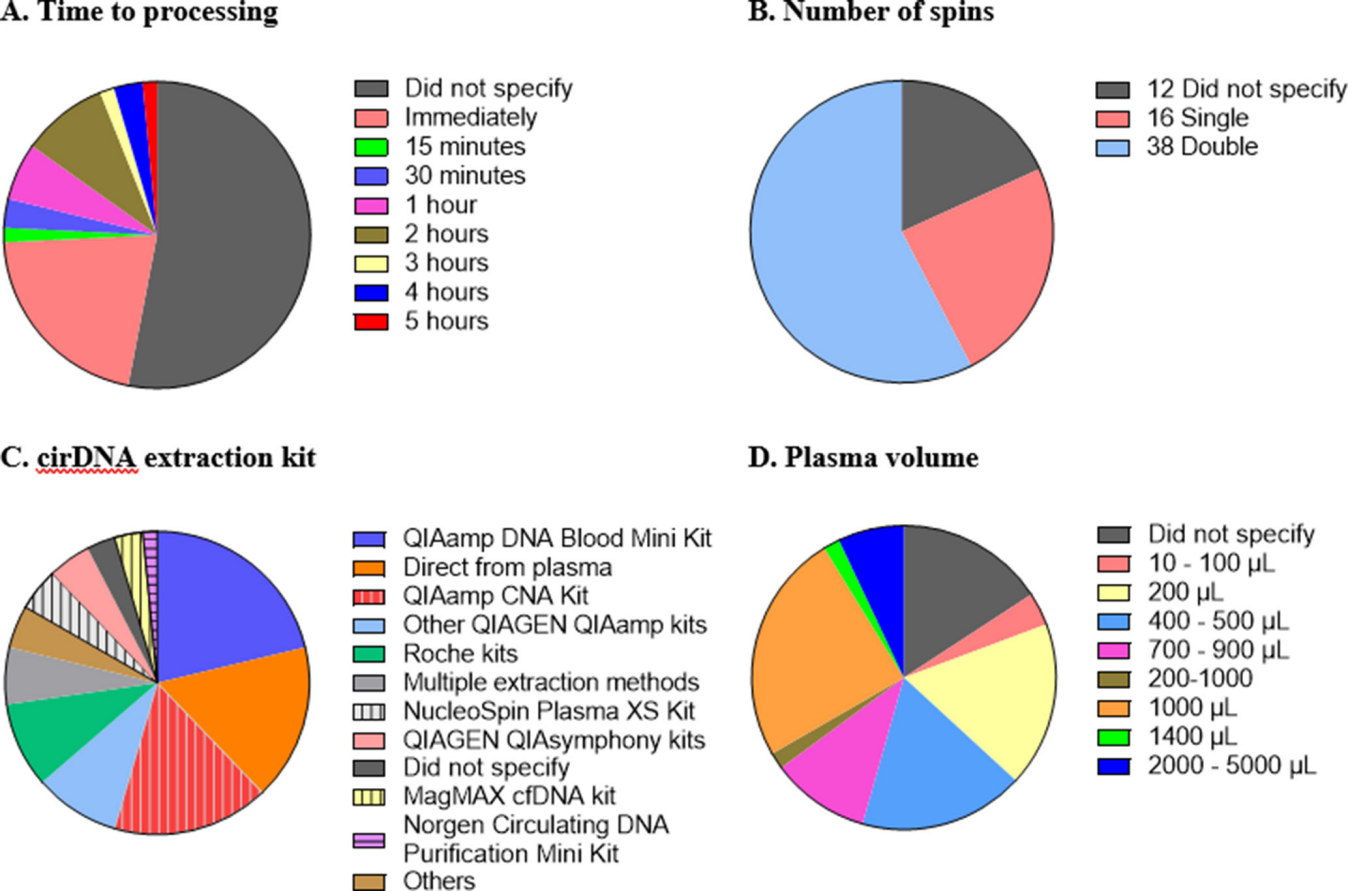
Technical aspects and protocol reporting in the 66 publications summarised in this review. (**A**) Interval time between blood collection and processing. (**B**) Number of centrifugations performed to obtain plasma samples from whole blood. (**C**) cirDNA extraction methods (specialised cirDNA kits are denoted by stripes). (**D**) Input volume of plasma into extraction (**D**).

While there is general agreement that standardised methodology and complete reporting of experimental parameters is desirable, consensus for a standardised set of protocols for cirDNA analysis has not been developed. There are numerous steps in cirDNA analysis, starting from blood collection all the way through to quantification, and variation at each step creates difficulty in comparing studies and a hindrance in translating biomarkers to the clinic. Here, we highlight some of the differences in the technical aspects of the 66 publications reviewed (Figure 1).

### Blood collection tube

A range of tubes for blood collection and plasma separation are available, including tubes stabilised to avoid leukocyte lysis. Most studies used EDTA tubes; however, 3 studies used heparin-based additives (Atamaniuk et al., 2010; Korzeneva et al., 2015; Sozzi et al., 2001), 15 studies did not specify the tube type (Supplementary file 1) and 2 were ambiguous (Czamanski-Cohen et al., 2013; Korabecna et al., 2011). The study by Meddeb et al., 2019 used Streck tubes when measuring the effect of circadian rhythm, and included a direct comparison to EDTA tubes, with no consistent differences observed.

### Time to blood processing

It is important that the time between blood collection and plasma processing is considered and reported as prolonged blood storage allows leukocytes to lyse and hence create genomic DNA contamination (Trigg et al., 2018; Warton et al., 2014). Different studies have used different ‘cutoff’ times such as 1 (Ohlsson et al., 2020; van der Drift et al., 2010), two (Brodbeck et al., 2020; Spindler et al., 2014) or 5 (Bu et al., 2020) hr. Out of the 66 studies, 35 (53%) did not provide any information regarding the duration between blood collection and processing. Of the 31 studies that do specify the time to processing, all fall within a reasonable time, with a maximum of 5 hr between blood collection and plasma separation (Figure 1A).

### Number of spins

A key step in blood processing for cirDNA extraction is centrifugation to separate the plasma from the red blood cells and leukocytes. Thirty-eight out of 66 studies (58%) centrifuged the blood samples twice, 16 studies (24%) did so once and 12 studies (18%) did not specify (Figure 1B). The role of the second spin is to minimise the potential for leukocyte contamination (Trigg et al., 2018), especially in case of inaccurate pipetting.

### cirDNA extraction

Sixty-four studies used a single extraction method while three utilised multiple methods to analyse the same cohort, including quantifying the cirDNA directly from plasma. In total, there are 11 studies that omitted the extraction step and quantitated cirDNA directly (Figure 1C).

Most studies used commercial kits to purify cirDNA. There are several kits available to specifically extract cirDNA such as QIAamp Circulating Nucleic Acid (CNA) Kit, NucleoSpin Plasma XS Kit, MagMAX Cell-free DNA Isolation Kit and Circulating DNA Purification Mini Kit. Only 37% of the 46 studies that involved a DNA extraction step used a kit specifically formulated for cirDNA. Using non-specialised kits results in substantially reduced cirDNA yields (Devonshire et al., 2014; Warton et al., 2018). For example, a comparison of the QIAamp CNA kit and the QIAamp DNA Blood Mini kit showed that the latter extracted less than one third of the DNA extracted by QIAamp CNA kit, with a bias towards high-molecular-weight DNA (Warton et al., 2018).

Out of these studies that did not use a dedicated kit, nearly half used QIAGEN kits (mostly QIAamp DNA Blood Mini Kit) and the remaining used a variety of other kits such as MagNA Pure LC DNA Isolation Kit-Large Volume. Some studies used various non-kit methods such as phenol-chloroform-based extraction (Jylhävä et al., 2011; Korzeneva et al., 2015; Tug et al., 2015) and in-house protocols (Tamkovich et al., 2005). Only two studies did not specify any information about DNA extraction method, nor if they did so.

### Volume of plasma used for extraction/quantitation

Both more (Alborelli et al., 2019) and less (Devonshire et al., 2014) efficient extraction of cirDNA has been reported with increasing plasma volume. About 40% of the studies used volumes below 1 mL for cirDNA extraction, down to as low as 50 µL (Tug et al., 2015; Figure 1D). Some of the low starting amounts are likely due to kit configuration, for example, the QIAamp DNA Blood Mini Kit specifies 1–200 µL of sample input. This again highlights the importance of using the appropriate kit to extract cirDNA.

### Quantitation method

The quantitation methods were predominantly qPCR, which remains the ‘gold standard’ for cirDNA quantitation (just over 50% of studies), followed by Qubit. Other means of quantitation were PicoGreen, cell death detection ELISA, SYBR nucleic acid gel stain, DNA DipStick TM Kit and GB genetic human DNA assay.

It is apparent in this analysis of 66 studies that there remain major inconsistences in the methods used to extract and analyse cirDNA. This not only raises questions about the validity of individual results but makes comparing studies and evaluating results difficult.

### Conclusion

**Figure 2.**
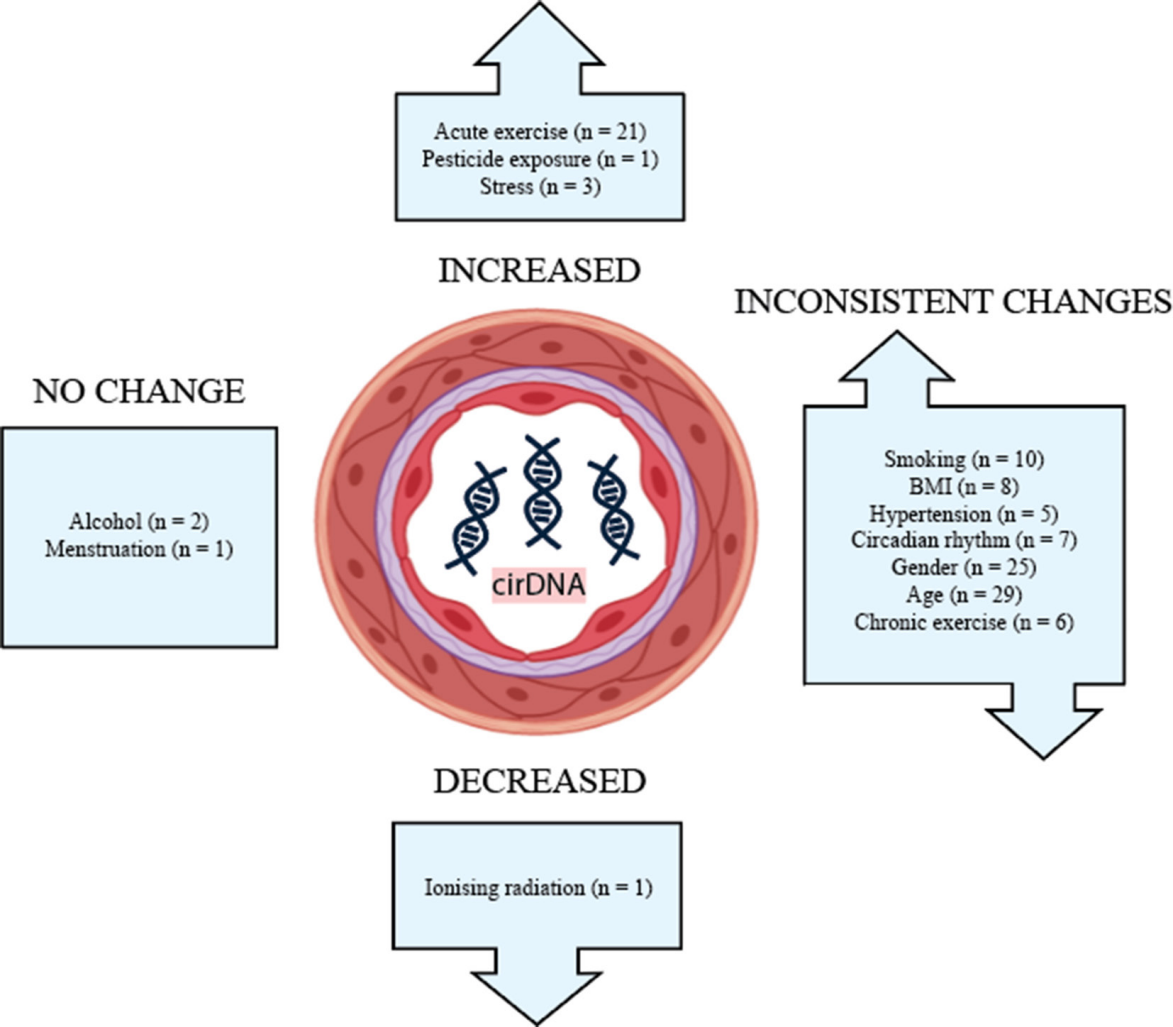
The effect of biological and lifestyle factors on blood plasma cirDNA concentration in healthy individuals and patients with various diseases and treatments.

The effects of lifestyle and biological factors on cirDNA are summarised in Figure 2. Despite technical and methodological variation between studies, acute exercise has consistently been shown to produce a robust but transient increase in cirDNA. The effect of exercise is very pronounced, with increases in cirDNA concentration that dwarf the rises reported in some disease states. In most exercise regimes, cirDNA returns to baseline level within a short amount of time (30–60 min); however, rigorous exercise induces a slower decline, with a measurable increase persisting at 48 hr (Margeli et al., 2005). Furthermore, the lowest level of exercise required for a measurable increase in cirDNA is not known, thus we propose limiting physical activity prior to a blood draw as a strategy to decrease baseline variation and improve capacity to detect differences between study groups. In line with this, study methods should report whether participant physical activity was controlled for.

Alcohol use and menstruation appear to have a negligible effect on cirDNA levels; however, the number of studies is small. Most other biological and lifestyle factors have been reported to both increase and decrease cirDNA levels. Better-designed and disaggregated studies are required to confidently rule in or out their effect.

Addressing each of these factors to accurately answer how they impact cirDNA level would require a very large study with a substantial number of participants and multidiscipline expertise to ensure that each factor is investigated and controlled properly. If this type of study was to be conducted, the best practice should be applied. Blood should be collected in a single-tube type, with short and consistent time between blood collection and centrifugation. Double centrifugation and specialised cirDNA kit for extraction should also be used.

A step towards obtaining more reliable data is choosing methods appropriate for cirDNA analysis, as well as careful sample handling and detailed reporting of protocols. Transparency in disclosing the way samples are processed and analysed may be aided by the development of reporting guidelines analogous to the Minimum Information for Publication of Quantitative Real-Time PCR Experiments (MIQE) guidelines (Bustin et al., 2009).

There remain large knowledge gaps in how common biological and lifestyle factors impact cirDNA. An understanding of cirDNA dynamics outside of a disease context is important for establishing accurate baseline levels and developing sensitive clinical tests that minimise the confounding effect of healthy variation.

## Additional files

Supplementary file 1. Impact of biological and lifestyle factors on cirDNA level.
